# Conservation and divergence of *Starch Synthase III* genes of monocots and dicots

**DOI:** 10.1371/journal.pone.0189303

**Published:** 2017-12-14

**Authors:** Bhavya Priyadarshini Mishra, Rajeev Kumar, Amita Mohan, Kulvinder S. Gill

**Affiliations:** 1 Department of Agricultural Biotechnology and Molecular Biology, Dr. Rajendra Prasad Central Agricultural University, Pusa, Bihar, India; 2 Department of Crops and Soil Sciences, Washington State University, Pullman, United States of America; National Institute of Plant Genome Research, INDIA

## Abstract

Starch Synthase (SS) plays an important role in extending the α-1,4 glucan chains during starch biosynthesis by catalyzing the transfer of the glucosyl moiety from ADP-glucose to the non-reducing end of a pre-existing glucan chain. SS has five distinct isoforms of which SSIII is involved in the formation of longer glucan chain length. Here we report identification and detailed characterization of ‘true’ orthologs of the well-characterized maize *SSIII* (*ZmSSIII*), among six monocots and two dicot species. *ZmSSIII* orthologs have nucleotide sequence similarity ranging from 56–81%. Variation in gene size among various orthologs ranged from 5.49 kb in *Arabidopsis* to 11.62 kb in *Brachypodium* and the variation was mainly due to intron size and indels present in the exons 1 and 3. Number of exons and introns were highly conserved among all orthologs however. While the intron number was conserved, intron phase showed variation at group, genera and species level except for intron 1 and 5. Several species, genera, and class specific *cis*-acting regulatory elements were identified in the promoter region. The predicted protein size of the SSIII orthologs ranged from 1094 amino acid (aa) in *Arabidopsis* to 1688 aa in *Brachypodium* with sequence identity ranging from 60%-89%. The N-terminal region of the protein was highly variable whereas the C-terminal region containing the Glycosyltransferase domain was conserved with >80% sequence similarity among the orthologs. In addition to confirming the known motifs, eleven novel motifs possibly providing species, genera and group specific functions, were identified in the three carbohydrate binding domains. Despite of significant sequence variation among orthologs, most of the motifs and their relative distances are highly conserved among the orthologs. The 3-D structure of catalytic region of SSIII orthologs superimposed with higher confidence confirming the presence of similar binding sites with five unidentified conserved regions in the catalytic (glycosyltransferase) domain including the pockets involved in catalysis and binding of ligands. Homeologs of wheat *SSIII* gene showed tissue and developmental stage specific expression pattern with the highest expression recorded in developing grains.

## Introduction

Starch is the most significant reserve of carbon and energy in plants. It is distributed widely among different plant species and constitutes a major source of calories (up to 50%) in the human diet [1]. As a raw material, it has great utility in various industries leading to the production of over 600 commercial products for both food and non-food uses [2]. Starch synthesis in higher plants involves a series of biosynthetic enzymes, including ADP-glucose pyrophosphorylase (AGPase), Starch Synthases (SS), branching enzymes (BE), debranching enzymes (DBE) and disproportionation enzymes [3]. Of these enzymes, Starch Synthase is involved in elongating α-1,4 glucan chains by catalyzing the transfer of the glucosyl moiety from ADP-Glucose to the non-reducing end of a pre-existing glucan chain through α -1,4-glucosidic linkages.

Based on the amino acid sequence similarity, five distinct classes of SSs genes have been identified in higher plants: Starch Synthases I-IV (SSI, SSII, SSIII and SSIV) and granule-bound SS (GBSS) [4]. These genes are highly similar in the C-terminal region, spanning ~450 amino acid residues comprising the catalytic and starch-binding domains, but have significantly variable N terminal region with SSIII having the longest N-terminal arm [4]. The sequence and the corresponding functions of the five classes of SS are broadly conserved among plants.

Among the five classes of the SS genes, the *SSI* is primarily responsible for the synthesis of the shortest chains whereas the *SSII* lengthens the short chains synthesized by *SSI*. The *SSIII* then produces longer length chains extending between the short chain polysaccharide clusters [5]. *SSIV* predominantly expressed in the leaves and there is low sequence identity with the Starch Synthases expressed in the endosperm [6],[7] whereas GBSS is mainly responsible for amylose synthesis. Although, the SS isoforms have distinct role in the synthesis of amylopectin, interaction and co-expression of isoforms were also reported. In maize, duII 1(*du1*) mutant characterized by loss of SSIII activity was also accompanied by loss of BEII activity [8]. *SSIII* loss of function is associated with the reduction in the proportion of the very long chains (dp>50) and slightly reduced gelatinization temperature in maize and barley [9] which is depicted by the shrunken phenotype the seeds. Suppression of *SSIII* in rice confers an increased amylose phenotype, with a reduction in the proportion of very long chains in amylopectin (>DP30), and slightly reduced gelatinization temperature [10]. Two independent null mutations in the *AtSSIII* locus were identified and were found to cause changes in the structure of the amylopectin in leaf starch and in the degree of starch phosphorylation [11]. Inspite of the fact that, there are few reports on characterization of SSIII in higher plants, the possible regulatory critical role of SSIII makes this an ideal candidate for further study.

True ortholog for *SSIII* gene are ubiquitous in plant kingdom with similar structural features and may play spatio-temporal/different physio-biochemical roles. *SSIII* loss of function is associated with the reduction in proportion of the very long chains(dp>50) and slightly reduced gelatinization temperature in maize and barley [12] and have shown similar effects in other dicots. Therefore, the focus of this study is to look at *SSIII* in the context of structural and functional features conserved across several taxa which may be responsible for the similar phenotypic effects.

Structural and functional conservation of the gene is important to reveal the important regions indispensable to its function as well as the variation unique to genera or species. With respect to the structural organization of SSIII, Gao et al. (1998) concluded that the maize SSIII sequence contained four distinct regions: a putative transit peptide region, a du1-specific N-terminal region, a central region homologous to other class III SSs, and a C-terminal region that contained the catalytic domain. The specific contributions of either the N-terminal variable region or the central SSIII-specific regions to the catalytic properties of the enzyme are unknown. However, the C-terminal region of maize *SSIII* expressed in *E*. *coli* was capable of catalysis, demonstrating that residues essential for catalysis are not located outside of the C-terminal conserved domain [4]. In dicots, this N-terminal region does not contain repeat motifs and is truncated Thus, a cross-species analysis of SSIII gives a foundation for investigating SSIII in crop plants where it is not well studied.

Among the enzymes involved in the pathway of sucrose to starch in wheat endosperms, soluble Starch Synthases (SSS) is the most sensitive to high temperature [13–15], and it has an unusually low optimum temperature for maximum activity. Heat stress decreases the activity of SSS in wheat, reducing grain growth and starch accumulation [16]. Even short periods of episodic temperature over 30°C slows starch accumulation principally due to heat induced denaturation of SSS. Maize SSIII activity was reduced by approximately 50% after brief incubation at 45°C, suggesting a role in reduced starch accumulation during growth at high temperature [17].

Considering the climate change and increasing trend in the temperature across the world, understanding the structure of SSIII with respect to genome organization, identification of domains and motifs involved in maintaining the catalytic properties and 3-D functional structure is important. Therefore, here we are reporting the comparative structural, functional and evolutionary conservation and differences in the *SSIII* gene among the species which were selected based on their agronomic importance, sequence information availability, plant anatomy (monocots vs. dicots) and diverse phenotypes.

## Materials and methods

### Identification of true orthologs

The *Starch Synthase III* gene is functionally well-characterized in maize thus the maize cDNA sequence was used as a reference to identify ‘true’ orthologs in eight plant species including six monocots and two dicots. ‘True’ orthologs were identified following the procedure described in Dhaliwal et al. (2014). Briefly the following criteria had to be met in order, for a sequence to be called a ‘true’ ortholog: 1. highest level of query coverage and sequence identity along the cDNA sequences; 2. the domains and motifs which were present along the protein used as reference sequence should also be present in orthologous sequences; and 3. equivalence in comparative size and distance among orthologous sequences. The cDNA and the full length genomic sequences for four monocots including rice (*Oryza sativa)*, barley (*Hordeum vulgare*), Sorghum (*Sorghum bicolor*), and Brachypodium *(Brachypodium distachyon);* and for two dicots including Arabidopsis *(Arabidopsis thaliana*) and Soybean (*Glycine max*) were identified from NCBI (http://www.ncbi.nlm.nih.gov) and Phytozome (http://www.phytozome.net/) database. The identified wheat ortholog of the SSIII gene was used to identify and assemble full-length genomic sequence of wheat (*Triticum aestivum*) SSIII from the “draft assembly of gene rich regions” of Chinese spring at the Cereals DB database (http://www.cerealsdb.uk.net/cerealgenomics/CerealsDB/search_reads.php). A consensus sequence was generated using the top hit contigs with the help of cap3. The procedure was repeated to obtain the contigs. The selected contigs were aligned using the “Align X” module of the *Vector NTI Advance*^*TM*^11.0 and the gaps were identified. The flanking sequence of the gaps were again used as query to search the database and obtained additional contigs until the gaps were filled. The procedure was repeated till the overlapping contigs were obtained and were assembled into a full-length genomic copy of the *TaSSIII*.

### Comparative analyses of gene structure

The exon/intron boundaries along with the translation start and stop sites was marked on the genes by aligning the cDNA with the corresponding genomic sequence using the “Align2 algorithm” of the “BlastN tool (http://www.ebi.ac.uk/Tools/msa/clustalw2/). The exons in different species were marked with reference to maize exons (depicted with same color as of corresponding maize exon). Intronic phase distribution was marked with respect to the location of an intron insertion relative to a codon. A phase distribution of 0 marks an intron insertion between two codons, phase 1 is insertion after the first nucleotide of a codon and phase 2 is insertion after the second nucleotide. Ka/Ks values defining the magnitude of non-synonymous to synonymous substitu- tions in various gene orthologs were calculated using the MEGA 6.0 software.

### Phylogenetic analysis

The evolutionary relationship among different Starch Synthase genes in the studied taxa was established using the MEGA 6 software on the predicted protein sequences. The phylogenetic analysis was carried-out with Maximum Likelihood method of distant matrix, which computes evolutionary distance using the Poisson correction method. The distances are in units of the number of amino acid substitutions per site. Bootstrap re-sampling with 1000 replicates was used to build unrooted phylogenetic tree and to infer the relationships.

### Predicted protein analysis of the SSIII orthologs

Predicted SSIII amino acid sequences of the eight species were aligned using ‘ClustalW’ to generate a consensus sequence. The maize amino acid sequence was used as a reference for the sequence comparisons among monocots and dicots. Consensus sequence was generated by retaining the aa with the maximum frequency of appearance across the species at the position. In the absence of a consensus at a position, maize aa was used. The sequence insertions present in one or more species were also included in the consensus sequence. Protein sequences of the eight species were then compared individually with the resulting consensus sequence to make the similarity analysis (S1 Fig). A scale of 1–6 for monocots and 0–2 for dicots was used to depict the respective aa similarity with the consensus sequence. A score of 6 for monocots indicates a conserved amino acid at a particular position in all 6 monocots whereas a score of 1 indicates that the residue is present in only one species. Similarly, for dicots, a score of 2 indicates conservation of the residue in both species. The already reported domains and motifs annotation of the consensus sequence was predicted by Conserved Domain Database (CDD) (http://www.ncbi.nlm.nih.gov/Structure/cdd/wrpsb.cgi).

### Functional analysis of the catalytic domain of SSIII

Predicted SSIII protein sequences of all the species were used to generate 3-D structure models on PHYRE2-Protein HomologY Recognition Engine (www.sbg.bio.ic.ac.uk/phyre2/;) using the Normal mode for modeling with default settings. The search engine is based on SCOP and PDB, and HMM-HMM alignment techniques for a remote homology detection. The protein model generated by the PHYRE2 was used to predict the ligand-binding sites by the 3D Ligand Site software? (http://www.sbg.bio.ic.ac.uk/3dligandsite;). The 3D Ligand Site predicted binding sites based on the comparison of query structure with a binding site library, which is extracted from protein–ligand complexes, to select similar structures followed by superimposition of the ligands on to the query structure to identify the binding sites. The ligand binding analysis identified three–five clusters in all the species. The cluster having the maximum number of ligands was selected for the prediction of the ligand binding in the target sequence. The models obtained from the PHYRE2 engine were selected based on their energy and quality parameters, evaluated using the SAVES (The Structure Analysis and verification Server; https://services.mbi.ucla.edu/SAVES) and Swiss-Model servers (swissmodel.expasy.org/docs/structure-assessment).

### Promoter analysis

Using the Phytozome database (https://phytozome.jgi.doe.gov/pz/portal.html), a 500-1000bp sequence upstream of the translation start site was obtained for each of the species. These sequences were used to find the regulatory element using the ‘Plantcare’ software with default paramaters (http://bioinformatics.psb.ugent.be/webtools/plantcare/html/).

### *Insilico* expression analysis in wheat

Expression analysis was carried on insilico using *WheatExp*, which is a homoeologue-specific database of gene expression profiles for polyploid wheat. Expression levels are calculated from RNA-seq datasets comprising multiple tissue and temporal developmental timecourses. cDNA of *SSIII* was used as query for BLAST search. The database comprise data of thirty RNA samples, corresponding to RNAs extracted in duplicates from five organs (root, leaf, stem, spike, and grain) at three develop- mental stages each from hexaploid wheat cultivar Chinese Spring. RNA-Seq libraries were constructed using the IlluminaTruSeq (Illumina, CA, USA). An average of 50 T 11 million paired-end reads per sample is mapped on the chromosome 3B scaffolds and used to reconstruct transcripts and estimate transcript abundance in units of fragments per kb of exon per million mapped reads (FPKM). Regions with FPKM values higher than zero is considered as expressed.

## Results

### Identification of ‘true’ orthologs

Since the *Starch Synthase III* gene was well characterized in maize, the *ZmSSII*I gene sequence was used as a reference to identify the true orthologs from eight plant species using the methods and criteria described in the ‘material and methods’ section. The longest reading frame of the predicted maize *Starch Synthase* (*ZmSSIII*) protein sequence was used as a query to identify its true orthologs. The true orthologous sequences from *Arabidopsis* and sorghum were retrieved from NCBI and that for barley, rice, *Brachypodium*, and soybean from Phytozome. (https://phytozome.jgi.doe.gov/pz/portal.html). Since the full-length sequence was not available for wheat, a complete orthologous genomic copy was assembled using 5X genomic sequence of Chinese spring (see materials and methods). CDS (AF258608.1) of wheat was used as a query against the draft assembly of gene-rich regions with an e value of e-50. Fifteen matches were obtained and assembled by CAP3 into 3 contigs. The contig with maximum length (2322) was used as a query to obtain 133 overlapping contigs. The contigs were assembled into full-length contig of 10144 bp. Exon-intron boundaries on assembled *TaSSIII* contig were marked using wheat CDS sequence and verified on Splign tool (http://www.ncbi.nlm.nih.gov/sutils/splign/splign.cgi?textpage=online&level=form

### Gene structure of SSIII

**Fig 1:**
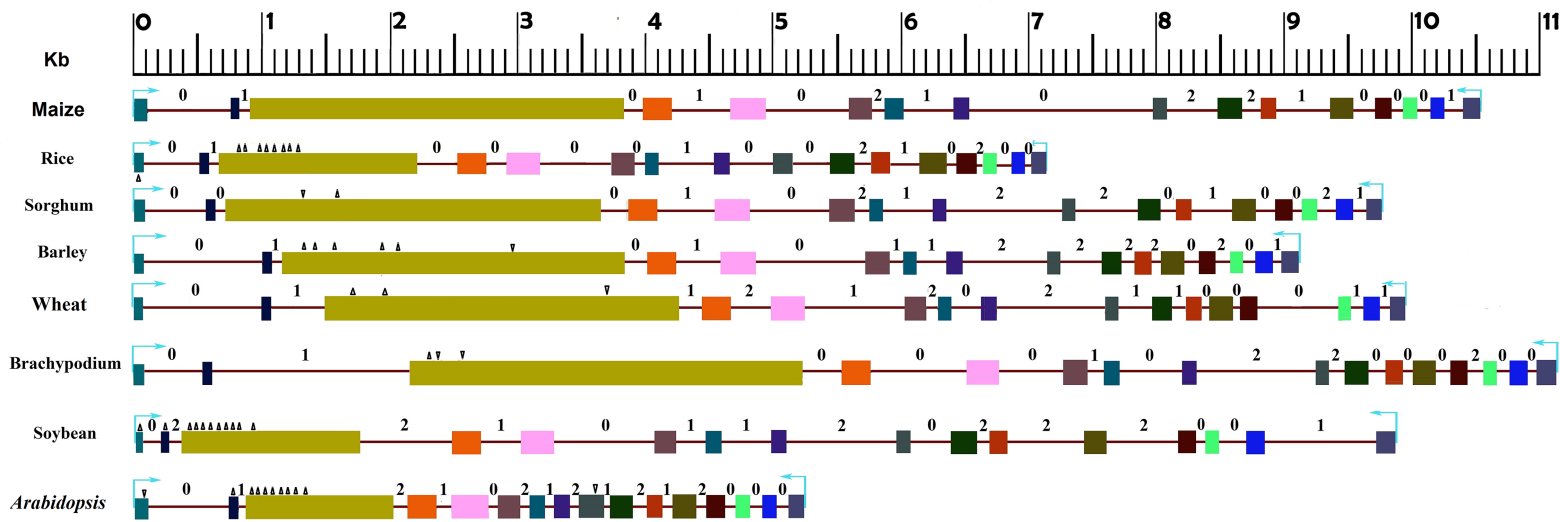
Structure of starch synthase gene. The translational start site to stop site is drawn to scale in six monocots and two dicots. Exons are represented by colored boxes and introns as lines. Each of the 16 exons in maize was marked by different color and corresponding sequences in different species was marked by the respective color in maize. Insertions (inverted black triangles) and deletions (upward black triangles) with respect to corresponding sequence in the reference gene are marked on different exons. Intron phases were marked by 0, 1 and 2 respectively.

The *SSIII* gene structure in monocots and dicots relative to that of maize (*ZmSSIII*) (Fig 1). The genomic copy of the *SSIII* gene is the longest in *Brachypodium* (11.6Kb)and the smallest in *Arabidopsis* (5.4Kb). Among monocots, rice had the smallest copy (9.4Kb) while in dicots, the smallest copy was present in *Arabidopsis* (9.4Kb). In general, the gene in monocots was larger than that of the dicots. These differences in gene size were attributable to the indels present mainly in the exon 1 and 3 and to the variation in the intron size. The exonic length of the gene in the monocots was larger than that in the dicots. The dicot species with the largest exome (Soybean, 3450bp) is smaller than the monocot carrying the smallest exome (rice, 4662kb). Even among the monocots, considerable variation was observed for the exonic length of the gene (Fig 1) (Table 1). The longest exon for the *SSIII* gene is in maize (5082bp) and the smallest was in rice (4662bp). The dicots showed little variation in the exonic length with the exonic length range from 3285bp in *Arabidopsis* to 3450bp in Soybean.

**Table 1:** Comparative lengths of exons and introns in base pairs (bp) among *SSIII* orthologs.

Species	1	2	3	4	5	6	7	8	9	10	11	12	13	14	15	16
	Exons															
Maize	99	70	2901	217	276	182	115	111	104	173	136	186	135	114	127	136
Rice	87	70	1542	216	273	180	115	111	105	173	131	186	134	114	129	136
Barley	90	70	2661	220	273	181	115	119	104	173	134	186	134	114	133	136
*Sorghum*	96	69	2931	217	276	182	112	110	104	174	136	186	135	128	127	136
Wheat	85	67	2664	220	273	181	115	119	104	173	134	186	132	114	133	133
*Brachypodium*	96	70	2940	216	273	181	114	119	104	174	131	186	134	114	141	136
Soybean	60	56	1367	217	273	178	112	110	108	173	132	185	138	120	130	138
*Arabidopsis*	117	64	1136	220	279	179	112	113	208	173	133	185	135	114	127	132
	**Introns**															
Maize	666	78	177	464	645	113	404	1466	408	133	399	125	99	103	111	-
Rice	439	81	299	169	567	64	418	381	352	136	236	104	92	81	76	-
Barley	926	82	196	344	858	106	249	660	310	91	79	115	98	89	73	-
*Sorghum*	464	80	210	465	634	115	402	920	467	126	311	141	83	130	97	-
Wheat	985	470	259	362	777	115	251	901	269	133	89	118	795	92	82	-
*Brachypodium*	2066	77	290	753	518	113	503	933	128	133	77	112	117	103	76	-
Soybean	2058	113	727	313	772	227	407	857	343	114	609	553	90	188	892	-
*Arabidopsis*	631	74	115	124	78	75	86	94	106	116	82	73	92	92	80	-

Both monocots and dicots have sixteen exons with the exon 3 being the largest and the exon 2 being the smallest. Length of most of the exons is relatively conserved except for exon 3 and exon 1. The exon 3 was highly variable with a size range of 1136bp in *Arabidopsis* to 2940bp in *Brachypodium*. This variation was mainly due to insertions and deletions. Both insertions and deletions were observed in monocots whereas mostly deletions were observed in dicots. Among monocots, compared to *ZmSSIII*, *BdSSIII* and *SbSSIII* have insertions of 39bp and 30bp respectively whereas *OsSSIII* and *HvSSIII* have cumulative deletion of 1359bp and 240bp respectively. The dicots have large deletions in the exon 3 relative to *ZmSSIII*; with *GmSSIII* and *AtSSIII* showing cumulative deletions of 1534bp and 1765bp respectively. The second most variable exon among the monocots and dicots was the exon 1. With respect to the reference *ZmSSIII*, soyabean has the smallest exon 1 with 39bp deletion followed by 12bp deletion in the rice gene. Barley has 9bp deletions, while 3bp deletions was present each in sorghum and *Brachypodium*. The longest exon 1 was present in *Arabidopsis* with an 18bp insertion.

The size of the exon 2 was perhaps the most conserved among the monocots whereas in dicots a deletion of 14bp in soybean and 6bp in *Arabidopsis* was responsible for the variation in the exonic length. Various exons showed significant differences for the extend of length conservation among plant species. The exon 4 was relatively conserved in length with small level of variation among monocots and dicots. The exon size was conserved among maize, sorghum and soybean whereas rice and *Brachypodium* have 1bp deletion and barley and *Arabidopsis* have 3bp insertion. Same trend was also observed for exons 5, 6, 8, 11, 13, 14 and 15. The exon 7 was conserved among dicots. The exon 9 of *Arabidopsis* has 104bp insertion in comparison to the reference gene in maize. Exons 10 and 12 were almost conserved in length among orthologs while, exon 16 was of same size among all monocots but showed 2bp insertion in soybean and 4bp deletion in *Arabidopsis*. The number of introns was conserved at 15 among all orthologs. Average total intronic length of the SSIII gene was found slightly greater than that of its coding sequence length. Relatively more variability was observed for the total intronic length among the studied species (64bp to 2066bp) in comparison to the exonic length (56bp to 2940bp). The intron 1 was the most variable (sd, 719) while, intron 13 showed the least variability (sd, 10.73). The total intron length was higher in dicots (5090bp) than the average intron length in monocots (4761bp). The overall noncoding size ranged from a minimum 3.5kb in rice to a maximum 6.0kb in *Brachypodium* among monocots compared to a minimum of 2.0kb in *Arabidopsis* and a maximum of 8.0kb in soybean among dicots.

### Intron phase evolution

Intronic phase distribution represents location of intron insertion relative to the location within a codon, as described in material and methods. Out of the 15 introns present in the gene, introns 1 and 5 have phase 0 in all the studied species (Fig 1). Intron 7 and 14 were conserved among all the studied species with single exception for each indicating species-specific evolution. Intron 7 has a phase distribution of 1 in all the orthologs except for *Brachypodium* (phase 0). Intron 3 and 12 showed genera specific conservation with phase 0 among the monocots and phase 2 among the dicots. Similarly, intron 14 has a phase distribution of 0 for all orthologs except in sorghum where it shows phase 2. The phase distribution of introns 4, 8, 10, 11 and 15 were relatively less conserved. Introns 4 and 15 followed the same trend of phase distribution of 1 except for rice and *Brachypodium* showing phase 0. Common phase distribution for intron 11 was also 1 except for barley and soybean where it was phase 2. Phase distribution of intron 8 in maize and rice was 0, but for the rest of the species it was 2. For intron 10, all the monocots and dicots have the phase 2 except in sorghum and *Brachypodium* where it was 0. The introns 2, 6 and 9 were having all three phases distribution among the studied species (Fig 1). The Ka/Ks values were calculated for all orthologs in a pair-wise comparison with the reference genome (“Material and methods” section) to determine relative effect of the sequence changes on the protein function (Table 2). The values ranged from 0.076 in *Sorghum* to 0.569 in *Arabidopsis* with an average of 0.391. The average value of 0.391 suggests that the gene has undergone positive purifying selection (Ka/Ks >1). The higher Ka/Ks ratio are putative cases of rapid evolution, and thus more likely to be associated with phenotypic changes.

**Table 2:** Ka/Ks values with respect to maize in different orthologs.

Species	Ka/Ks values						
Maize							
Wheat	0.320						
Barley	0.321	0.062					
Rice	0.358	0.35	0.343				
*Sorghum*	0.076	0.308	0.317	0.357			
*Brachypodium*	0.307	0.178	0.178	0.343	0.299		
Soybean	0.518	0.531	0.530	0.48	0.515	0.533	
*Arabidopsis*	0.544	0.565	0.565	0.538	0.542	0.569	0.404

### DNA sequence comparison

As expected, *ZmSSIII* coding sequence showed more similarity to its monocot orthologs than that of the dicots. It showed an identity of 93% to sorghum, 78% to both of barley and *Brachypodium*, 76% to rice, 69% to soybean and 66% to *Arabidopsis*. Maize exon7 showed maximum sequence identity with corresponding region in monocots ranging from 85% in barley and *Brachypodium* to 96% in sorghum; whereas exon6 exhibited maximum sequence identity with dicots ranging from 77% in *Arabidopsis* to 78% in soybean. With few exceptions exon2 has overall lowest sequence identity to corresponding sequence of studied species with lowest value of 47% for *Arabidopsis*. Overall, exons of maize have sequence similarity with monocots in the range of 78–96% compared to 76% identity in the dicots (Table 3).

**Table 3:** Percentage identity of exons in different species with reference to maize *SSIII*.

Species	Exons															
	1	2	3	4	5	6	7	8	9	10	11	12	13	14	15	16
**Rice**	72	65	70	76	81	81	87	73	83	79	79	78	71	74	79	74
**Barley**	67	72	66	73	82	81	85	78	88	84	87	83	73	78	78	77
***Sorghum***	90	90	92	95	96	96	96	94	93	94	94	92	91	94	91	96
**Wheat**	69	72	66	73	82	81	85	78	88	84	87	83	73	78	78	77
***Brachypodium***	69	71	65	79	80	81	85	84	90	83	83	80	73	78	75	80
**Soybean**	68	59	64	65	70	78	61	71	70	77	71	64	69	74	74	70
***Arabidopsis***	51	47	65	61	68	77	75	70	66	71	65	70	63	74	71	74

### Phylogenetic analysis

**Fig 2:**
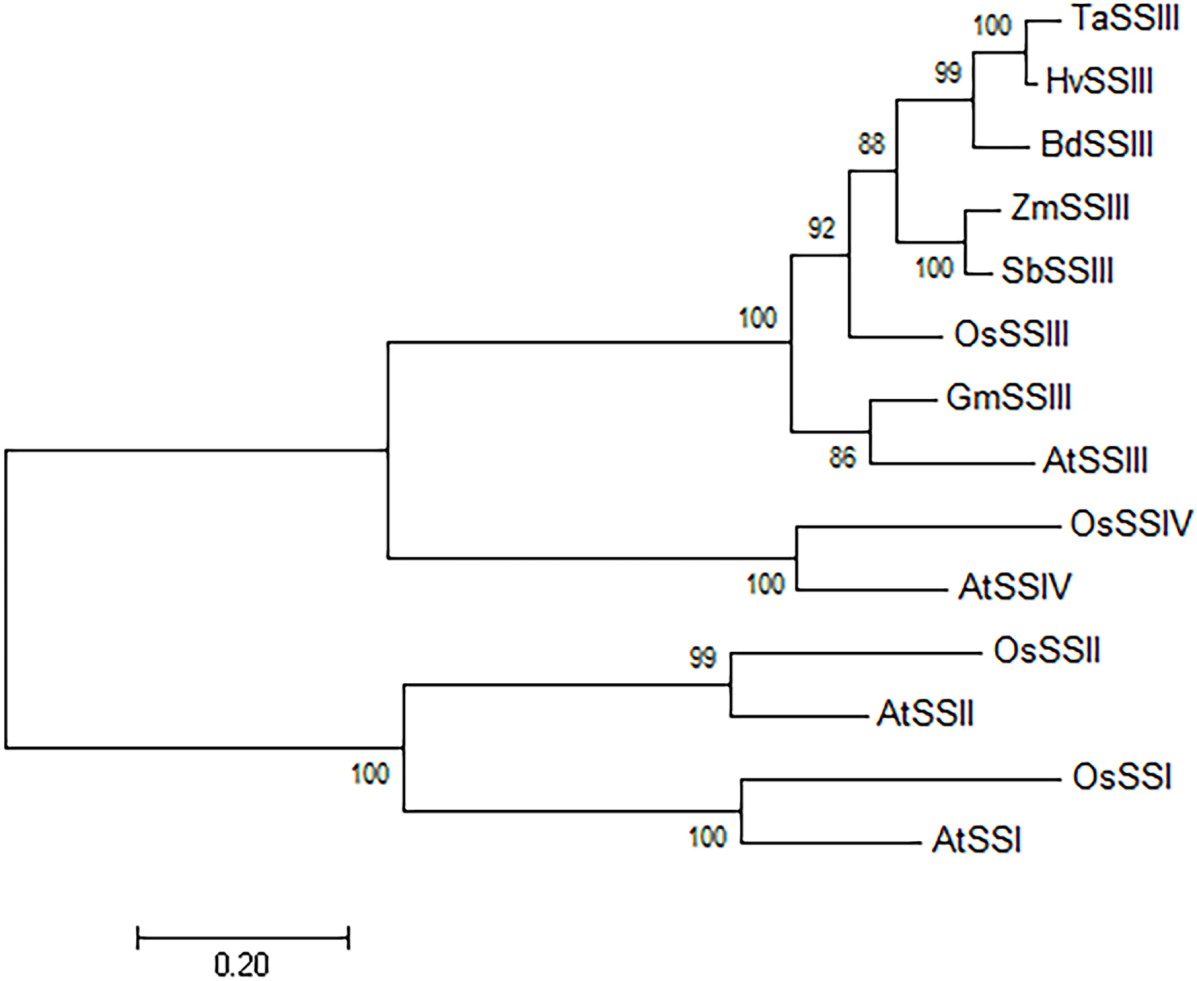
Phylogenetic tree. It is constructed using Maximum-likelyhood method showing evolutionary relationship between six monocots and two dicots. The bar at the base showed the nucleotide substitution per site.

For deciphering the evolutionary relationship of Starch Synthase genes among monocots and dicots, the predicted protein sequence of the SSIII from othologous species and other Starch Synthases (I, II, and IV) were used for the analysis. The *Arabidopsis* SS genes clustered into two groups, one consisting of SSI and SSII and another consisting of SSIII and SSIV (Fig 2). The SSIII cluster further diverged into two sub-clusters accounting for the divergence of the monocots and dicots. During the evolution, the rice gene diverged from the rest of the monocots that formed two groups with one comprised of maize and sorghum and the second with wheat, barley and *Brachypodium*. The *Brachypodium* gene further diverged from wheat and barley. As expected, closely related species showed less sequence changes as compared to the distantly related species. In sub-cluster I wheat and *Brachypodium* were more divergent than wheat and barley, maize accumulated more changes than rice than maize and sorghum. Among dicots *Arabidopsis* has acquired more sequence changes compared to soybean.

### Protein analysis

The size of the predicted SSIII protein ranged from 1094aa in *Arabidopsis* to 1686aa in *Brachypodium*. Compared to ZmSSIII, protein sequence similarity among orthologs ranged from 61 to 89%. Most of the mismatches and deletions were present around the N- termini whereas, the C-termini were conserved among the orthologs. Among the monocots, rice showed a deletion of 200 aa and was more closely related to the maize, sorghum, and *Brachypodium* genes than with that from wheat or barley (Table 4). Relatively the N-terminus of the dicot genes showed more aa deletions, for example, a 495aa deletion in *Arabidopsis*. This analysis showed that while there was higher sequence similarity in the SSIII-specific N-terminal and C-terminal catalytic domains between wheat and maize, the conservation was low in the region extending from the putative transit peptide to the SSIII-specific region. The monocot and dicot N-terminal region sequences showed marked differences in both length and homology, and there is only very weak evidence of repeats in the dicot sequences therefore, we refer to the region between the putative transit peptide and the SSIII-specific region as the N-terminal variable repeat region in the monocot sequences and as the N-terminal region in the dicot sequences.

**Table 4:** Percent identity of different domains of SSIII in comparison to maize.

Species	Catalytic domain	SSIII specific region (CBD)	Variable region
Rice	82	73	34
*Sorghum*	95	94	82
Barley	85	72	36
Wheat	84	72	39
*Brachypodium*	84	74	36
Soybean	77	58	25
*Arabidopsis*	75	56	23

**Fig 3:**
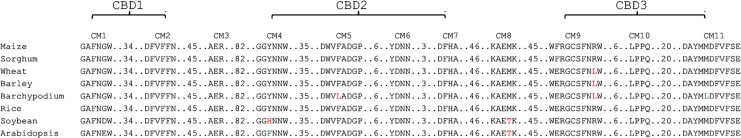
Novel conserved motif (CM) identified in the carbohydrate-binding domain (CBD) of starch synthase among monocot and dicot species. Conserved regions are in black, synonymous changes in green and non-synonymous changes in red. Number represents the distance between the two CMs in terms of amino acids.

The Conserved Domain search identified multiple carbohydrate binding domains (CBD) between residues 200 to 1250 (S1 Fig). Among monocots, the three CBDs were present between residues 709 to 1228 except in rice where the domains were present between residues 333 to 758. In dicots, the CBDs were found to be present between residues 205 to 689. The size of the three CBDs was conserved among various orthologs with the maximum variation in CBD2. The distance between the CBDs was highly conserved among the orthologs (Fig 3). As protein sequence level, CBD3 was the most conserved with 60% similarity among orthologs followed by CBD2 with 45% similarity. The CBD 1 was the least conserved with 38% similarity among the orthologs.

The Glycosyltransferase domain was conserved among all the species. Compared to the maize gene, sequence similarity ranged from 95% (Sorghum) to 75% (*Arabidopsis*). Within catalytic core is the conserved starch catalytic domain (Pfam PF08323) and the glycosyltransferase 1 domain (Pfam PF00534) characteristic of the GT5 glycosyltransferase superfamily according to CAZY. The starch catalytic domain is found in glycosyltransferases that use only ADP-glucose as substrate. The glycosyl transferase 1 domain is found in proteins that transfer UDP, ADP, GDP or CMP-linked sugars to a variety of substrates, including glycogen, fructose-6-phosphate and lipopolysaccharides and is therefore not unique to GSs and SSs. Five conserved motifs are found in this region two of which encompasses the highly-conserved motifs i.e. KVGGL and KTGGL that plays role in catalysis and/or substrate binding. The distance between these motifs are also conserved in all the orthologous species.

### Identification of novel motifs and domains

In addition to the well-known motifs, 11 novel motifs (CM1-11) were found in the three CBDs of the SSIII gene. Both the relative motif size as well as the distance among the motifs were conserved among species (Fig 3). For the most part motif sequences were conserved among orthologs except for few synonymous and non-synonymous changes in CM4, CM5, CM8 and CM9. In general, more changes were seen in dicots. The CM9 showed a single aa non-synonymous change in wheat, barley and *Brachypodium* that appears to be monocot specific. Another non-synonymous change was seen in CM5 that was present only in *Brachypodium*. Sequence of the linker regions (AE) connecting various CBDs were also highly conserved among the orthologs. Both the distance as well as the sequence of the linker (**AE**R) between CBD1 and CBD2 were highly conserved. The same was the situation for the second linker region (K**AE**MK) connecting CBD2 and CBD3 except for a single synonymous change in dicots where the M is replaced by a T (Fig 3).

### Functional structure of catalytic domain of SSIII

With the objective to compare functional conservation of the protein, 3-D structures of catalytic domain of protein of the orthologs under study are obtained, as described in Materials and Methods. Models obtained from PHYRE2 engine were selected on the basis of their energy and quality parameters checked from SAVES [(The Structure Analysis and verification Server) https://services.mbi.ucla.edu/SAVES] and Swiss-Model server (swissmodel.expasy.org/docs/structure-assessment). The SWISS-MODEL template library provided the annotation of quaternary structure and essential ligands and co-factors to allow for building of complete structural models, including their oligomeric structure.

**Fig 4:**
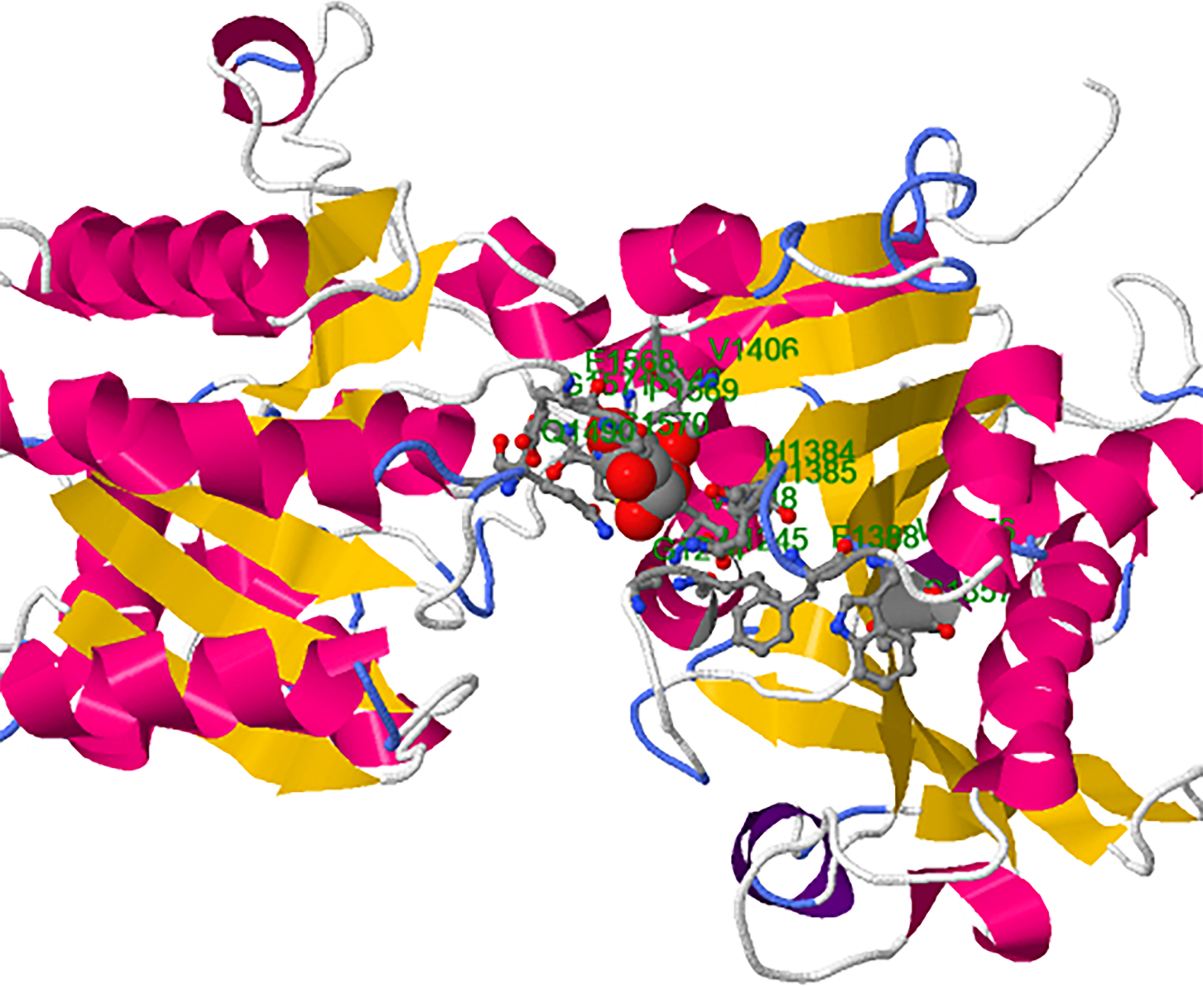
Three-dimensional structure of catalytic region of SSIII in wheat. The amino acids and their position in the protein involved in ligand binding are shown in green. Red sphere depicts heterogen involved in ligand binding. Dark pink color coils are alpha helix and yellow arrows depict beta-sheets.

**Fig 5:**
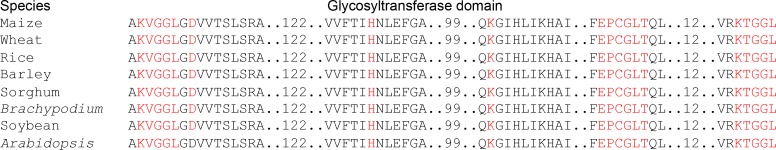
Conserved regions identified in the glycosyltransferase domain of SSIII. Residues involved in ligand binding are depicted in red. Number represents the distance between the conserved regions of domain.

Based on the 3-D structure, the catalytic portion of SSIII consists of 20 β-sheets and 24α-coils (S2 Fig). A homology model for the C-terminus of SSIII of orthologous species was built by threading its sequence onto the 3D structure of *Agt*GS (Fig 4). The two different α-β-α *Rossmann* domains of the GT-B family are apparent as is the large cleft which contains the ADP-binding pocket towards the C-terminal side. Amino acid residues that take part in catalysis and/or substrate-binding are depicted as an ADP-binding pocket on the model. Some of the largest and perhaps the most conserved features of TaSSIII are the irregular loop structure KVGGL located between 1190 to 1200 residue, theβ-sheet containing irregular loop ITRLT located around 1440 residue and the irregular loop FEPCGLT that is consisted of β-sheets and α-coils located between 1520 to 1530 (Fig 4). The ITRLT and the FEPCGLT ADP binding pockets were found in the highly-conserved regions of the C-terminal portion of the protein (Fig 5).

Ligand binding analysis was performed to predict binding of various ligands to the Glycosyltransferase domain. Three predicted amino acid clusters were obtained for binding of ADP (Adenosine Diphosphate), G6P (Glucose-6-Phosphate), PLP (Pyridoxal-5-Phosphate) and GLC (Alpha-D-Glucose) ligands (Fig 4). Cluster I with the maximum number of ligands was chosen for the analysis which showed that most of the amino acids involved in the ligand binding are conserved among the orthologs (Fig 5). Most ligand binding was towards the N-terminal amino acids of the catalytic portion of the Glycosyltransferase domain. Thirty-five ligands were identified in the cluster consisting of 7 ADP, 2 CFF, 1G6P, 13 PLP and 12 GLC as heterogens. Eleven aa including LYS, ASP, HIS, ARG, LYS, GLU, PRO, CYS, GLY, LEU and THR are involved in the ligand binding. All the ligand-binding sites are present in the irregular loop of the domain.

**Fig 6:**
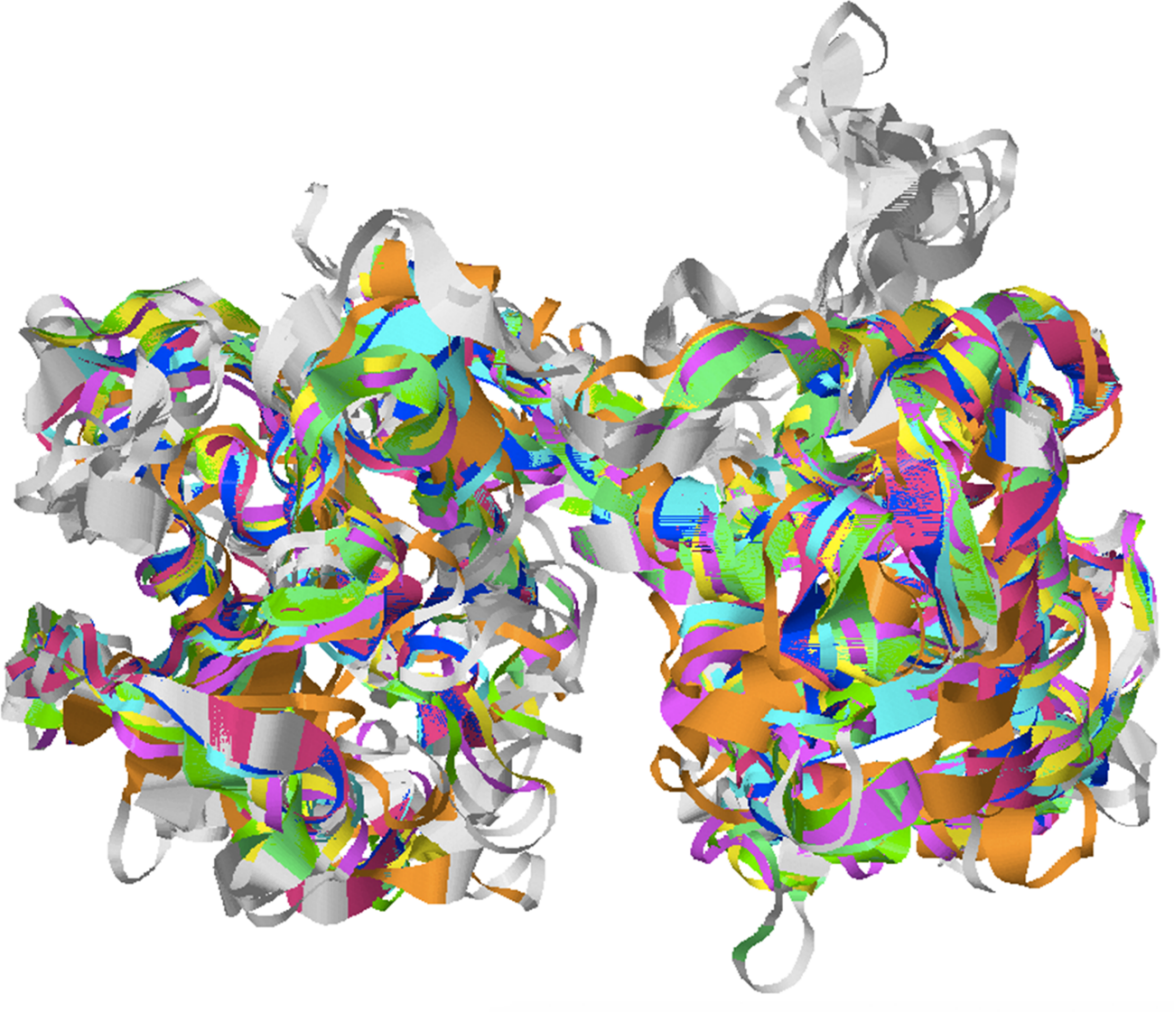
Superimposed 3-D structure of catalytic domain of SSIII among all the orthologs. Green color depicts *Arabdopsis*, yellow- soybean, blue- barley, red- wheat, orange- sorghum, pink- maize and cyan- *Brachypodium*. Grey color represents the unaligned portion of the superimposed structure of domain.

The superimposed structure of the catalytic domains of the orthologs showed that the 3-D conformation of two different α-β-α Rossmann domains of the GT-B family, apparent as the large cleft containing the ADP-binding pocket, superimposed well except for few regions (depicted as gray in Fig 6). Residue-by-residue mapping data also showed a high degree of similarity in the active binding sites of all of these proteins. It was found that the root mean square deviation was the highest in case of Sorghum but the Q-Score (which measures the quality of an alignment) was the highest in case of Soybean (Table 5).

**Table 5:** 3-D structural analysis of superimposed regions of catalytic domain among all the orthologs.

Species	N_RSE_^a^	N_SSE_^b^	RMSD	Q-Score
Maize	444	26	1.66	0.56
Sorghum	443	32	2.89	0.38
Wheat	443	32	1.50	0.59
Barley	443	34	1.36	0.62
*Brachypodium*	443	32	1.35	0.62
Rice	456	30	1.45	0.58
Soybean	456	30	1.15	0.63
*Arabidopsis*	445	31	1.38	0.60

^a^N_RSE_(number of aligned residue)-328

^b^N_SSE_ (Number of aligned secondary structure)_-_16; Overall RMSD-2.53; Over all Q-Score-0.31

### Promoter analysis of the SSIII gene

For identifying the common regulatory regions in the SSIII proteins, promoter analysis was carried out in all the species. Around 500–1000 nucleotide sequence upstream of the ATG codon of the genes was retrieved using the Phytozome database (https://phytozome.jgi.doe.gov/pz/portal.html) and promoter elements were identified using the PlantCARE database. Compared to that of maize, the promoter sequence showed sequence identity of 46% with wheat, barley and *Arabidopsis*, 49% with rice, 45% with *Brachypodium*, and 47% with soybean. The highest sequence similarity of 75% was with that of sorghum. ARE motif, a cis-acting regulatory element essential for the anaerobic induction, was found in wheat, maize and *Brachypodium*. The CAT BOX, a cis-acting regulatory element related to meristem expression, was present in *Brachypodium*, *Arabidopsis*, and Sorghum but was missing in others. The CCGTCC-box, a cis-acting regulatory element related to meristem specific activation, was identified in wheat, *Brachypodium* and *Arabidopsis*. Interestingly the ERE (ethylene-responsive element) was present in wheat however, it was absent in all other monocots and dicots. Two HSE (heat responsive element) elements were also present in wheat and *Brachypodium* but not in any of the other species. Whereas the light responsive element G-box is present in all the species but was absent in rice and soybean. Several putative TATA box and CAAT box sequences are also present in all of the species but are absent in rice (S1 Table).

### *Insilico* expression pattern of SSIII homeologues in wheat

**Fig 7:**
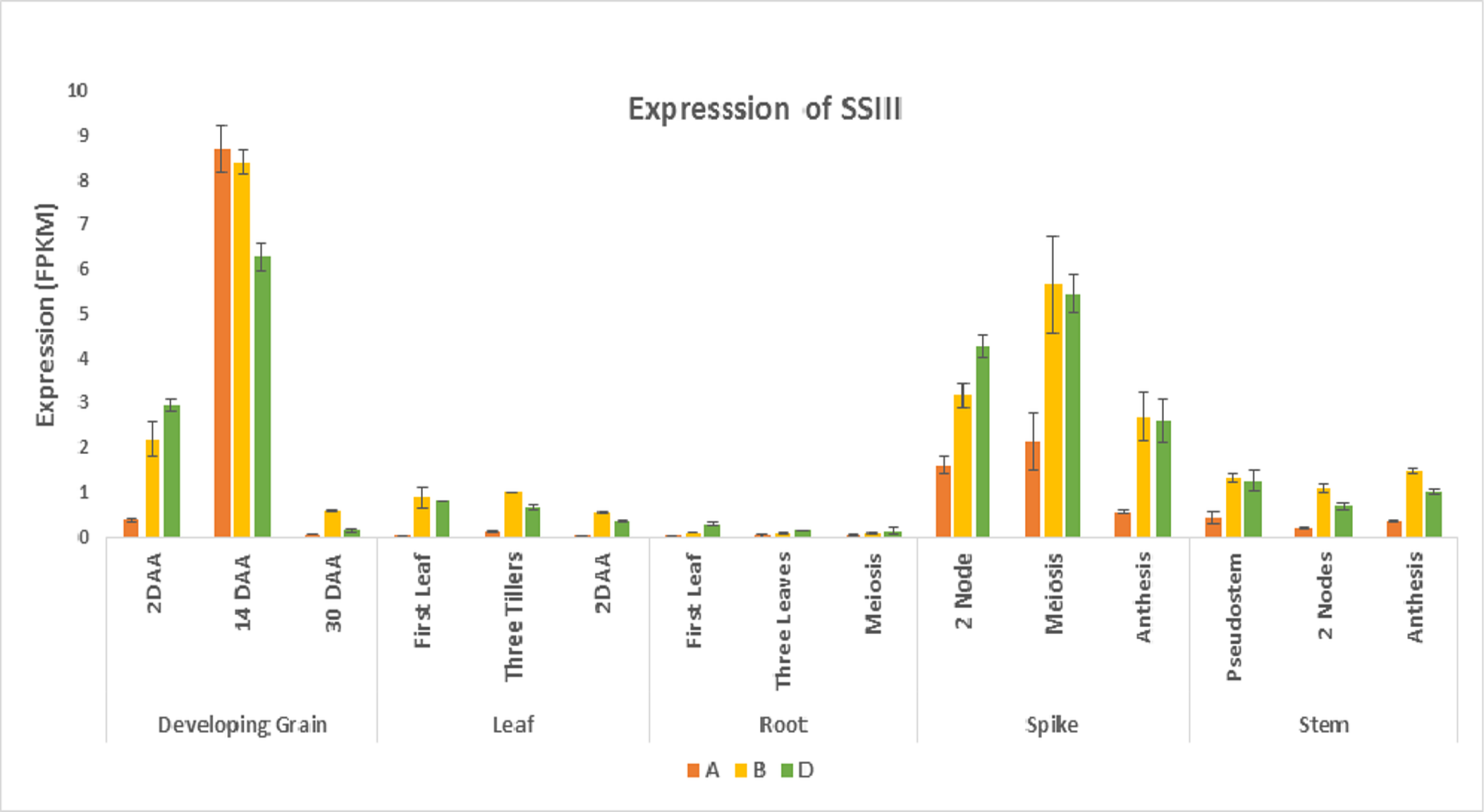
Expression profile of *TaSSIII* in various tissues and development stages.

For homeologous triplets of SSIII gene, spatiotemporal differences of expression patterns were observed. All the three copies exhibited highest expression in developing grain with increased expression at 14 DAA was highly expressed in developing grain. Starch Synthase III gene expression is dominated by the A and B homeologs during grain and spike development respectively, whereas the D copy exhibited less but consistent expression. Lowest expression of all the homeologous copies was recorded at 30 DAA of developing grain. The expression level was drastically low in leaf and stem while negligible expression was recorded in root. Lesser decline in the expression the SSIII gene was observed during the spike developmental phase, with all the homeologs expressing simultaneously (Fig 7)

## Discussion

In plants, starch plays an important role by serving as a source of energy and available carbon. Of the various starch biosynthetic enzymes, Starch Synthase III is mainly involved in extending the α-1,4 glucan chains by catalyzing the transfer of the glucosyl moiety from ADP-glucose to the non-reducing end of a pre-existing glucan chain. The amount of amylose and amylopectin in starch is the determinant of its quality which is directly affected by this enzyme. Heat stress affects grain quality of cereals by changing activities of various carbohydrate metabolizing enzymes including the heat labile soluble Starch Synthases [13–17]. Thus, understanding the structural and functional conservation of the Starch Synthase gene is important to reveal the important regions indispensable to its function as well as the variation unique to genera or species. This knowledge may also be useful to engineer an ideal starch synthase enzyme.

Finding true orthologs is particularly important for a multi-gene family as other members of the family share common domains and motifs that may play spatio-temporal/different physio-biochemical roles. A criteria was developed to find true functional orthologs of a member of a multi-gene family by taking the ABCB multi-gene family as an example [18]. Using the criteria, true orthologs of the maize Starch Synthase III genes were identified in five monocots and two dicot species. The maize starch Synthase III gene was taken as a reference as it was the best characterized gene among all plants [19]. The identified genes appear to be true functional orthologs because: 1. All the signature regions present in *ZmSSIII* including Carbohydrate Binding Domain and Glycosyltransferase domain were present; 2. The distances among the motifs and key domains were also conserved; and 3. There was a high level of protein sequence conservation among the orthologs, especially for the functionally important regions. Furthermore, analysis of all the soluble starch synthases from Rice and *Arabidopsis* showed that the carbohydrate binding domain is present only in the SSIII gene which may be attributed to the fact that it produces longer glucan chains (data not shown). This important domain was present in all the identified SSIII orthologs. Thus, the identified SSIII from the five monocots and two dicots represents true functional orthologs of ZmSSIII.

Intron number, location and phase are important features to study evolution of a particular gene. The gene structure variation among eukaryotic species is mainly due to the variation in the intron number and size. Gene structure with respect to number of introns and exons was highly conserved among the identified *SSIII* orthologs. The number of introns was conserved even in the common ancestor of monocots and dicots *Amborella trichopoda* (data not shown) indicating that the intron insertions occurred before the monocot-dicot divergency without much change after that. Although most of the intron phase was conserved among various orthologs supporting this conclusion, there were several exceptions to this as well. These species-specific and genera-specific intron insertions in the same general area is very interesting as it points to the presence of sequences or chromatin features that are relatively more prone to intron insertions. We however do not have a direct evidence to support this claim. Although the number and insertion site of introns were generally conserved, the size of many introns was very different explaining most of the size variation among orthologs.

Some variation in gene size was also due to the size of exon 3. The exon 3 accounts for about 50% of the expressed part of the gene and codes for the N terminal variable repeats region of the gene along with much of the SSIII-specific region. This indicates it’s being the recent insertion in the gene structure due course of evolution. The sequence similarity among the exons is also high except for the first three exons corresponding to the variable N-terminal region of the protein. This indicates relatively low selection pressure on exome coding N-terminal variable region which is not performing any catalytic function but may be performing some interactive role thus allowing relatively high rate of evolution. Comparison of the rest of exon among the orthologous species shows that they are relatively more conserved across the studied genera and corresponds to the distance and the location of conserved motifs in the C-terminal catalytic domain within exons.

In comparison to exome the introme of the gene contributes 1.4 times more in total length wherein intron 1 has significant contribution individually in comparison to others, which also exhibits much size variability. Size variation among orthologs of the gene is significantly due to variation in size of introns as it may be under relatively less selection pressure [20]. Total 15 introns were observed in each of the studied orthologs including monocots as well as dicots. Phase distribution of the introns differed significantly from random distribution of 33% of each wherein insertion at phase zero is 43%; phase one is 33% and phase two is 24% indicating more bias towards phase zero and least towards phase 2 (Fig 1). Dhaliwal et al., 2014 also reported bias in phase distribution of ABCB1 gene introns. However, the dicot specific introns are exhibiting almost random distributions to all three phases. Out of 15 introns only six (2, 6, 9, 10, 11 &14) are having all the three phases among the orthologs.

In general, the general domain and motif organization of the *SSIII* gene is conserved across the taxa possibly retaining the primary function over the course of evolution. SSIII protein has four distinct regions: The N-terminal putative transit peptide region, the taxa specific N-terminus region, the SSIII specific region (residues 771–1,239 of maize SSIII) and C-terminal catalytic region [21],[22]. The protein is deposited as complex crystalline discrete granules made of α glucans both in the leaf cell chloroplast (transient starch) and in amyloplasts of the storage plant tissue (storage starch) [23]. Less than 100 residue long putative transit peptide is for the cellular localization of the protein. The bioinformatic analysis of the transit peptide has revealed that the amino acid sequence of this region has preponderance of hydroxylated amino acids including Serine, Threonine, and Proline and lacks in acidic amino acids thus is hydrophobic [24]. This region also contains various highly conserved sequence motifs that play important role in interactions with other protein factors and taking helical shape while interacting with plastid membranes during import process of cellular localization. Some of these motifs are also present in related proteins thus identifying novel features that distinguish SSIII from other related proteins is very critical to identify true functional orthologs. The N-terminal region is just proximal of the transit peptide. The region confers regulatory role to the enzyme with respect to starch synthesis [25]. It consists of taxa specific N-terminal region and the SSIII specific region (S1 Fig and Fig 3). Former is the most variable region in terms of length and homology. Though the region is less conserved among taxa under study, a marked difference has also been observed between monocots and dicots wherein the monocots carry a larger size and more repeats in comparison to the dicots (Table 4). The specific repeat motifs in the region allow it to interact with other enzymes in the same metabolic pathways [26]. This region is absent in the dicots under study. The immediate proximal region is an SSIII specific region as it is present only in the SSIII isoforms. The region consists of three Carbohydrate Binding Domains (CBDs) separated by linker regions. The CBDs are non-catalytic in nature and belong to the carbohydrate-binding module (*CBM) 53* family [27]. It provides unique substrate binding ability to SSIII. In *A*. *thaliana* CBD2 has the highest starch binding affinity. This domain shows structural similarity to the CBD of GA-1 from *Aspergillus niger* [28]. An alignment of CBD sequences from CBM20s, CBM21s, CBM48s and CBM53s has indicated a high degree of conservation of a tryptophan and an aromatic residue in the starch binding site [29] equivalent to W366 and Y394 in *Arabidopsis*, the double mutation which led to a six-fold reduction in the affinity. These key conserved features were present in all the orthologs. Among the identified orthologs, the size of the three CBDs was found to be almost constant with relatively conserved sequences (Fig 3). The linker sequences among them were also constant. In maize, an equivalent SSIIIHD region showed binding affinity to SSI. A coiled-coil domain in the region could be responsible for both protein interaction and glucan binding, shown in lactobacilli, the tandem arrangement of CBDs is suited for disruption of starch structure [30]. In *A*. *thaliana* interaction between CBDs and C-terminal catalytic domain was shown to modulate the enzymatic activity [29] highlighting significance of its tandem structural arrangement in improving starch binding and increasing its concentration to active site of the enzyme thus improving enzymatic efficiency.

The highly-conserved C-terminal region of the protein possesses the residues that are essential for catalysis. This catalytic activity is self-contained as the C-terminal region of maize SSIII expressed in *E*. *coli* was capable of catalysis [4]. This catalytic region is highly conserved among all the SS isoforms. This is also conserved in Glycogen Synthase as and SSIII-CD from *A*. *thaliana* could successfully complement glycogen deficient mutant in *A*. *tumefaciens* [31]. It is 60 kDa in length and encompasses the complete amino acid sequence of the prokaryotic GSs (Denyer et al., 1999). The catalytic core comprises of starch catalytic domain and the glycosyltransferase 1 domain (GT1), characteristically falling into GT5 glycosyltransferase superfamily. Out of the two domain GT1 was found relatively more variable. It may be due to for meeting the requirements of specific texa/group with respect to variation in reaction mechanism in transferring nucleotide sugar to the substrates. The presence of GT-B fold consisting of two Rossmann like α-β-α domain separated by a deep cleft containing ADP binding pocket reflects the characteristic of “retaining-type” glycosyltransferase. G-X-G loop that faces the nucleotide binding cleft is responsible for ATP binding, though considered highly conserved was found absent in *At*SSIII. Occurrence of one of the two K-X-G-G-L motifs in the identified conserved regions of the domain (Fig 5) indicates its role in catalysis as well as substrate binding. It has been reported that KXGGL motif has a direct role in nucleoside-diphospho-glucose binding in *Escherichiacoli* GS, although only the glycine residues but not the basic side chain appears to be essential for enzymatic activity. This motif is highly conserved and may be playing important role in chain elongation specificity and the variable residue in this conserved motif may be responsible for preference of primer with specific length. In the present study valine amino acid residue was found at that place with respect to which intron position was also found conserved indicating its key role in metabolic activity. The analysis of secondary structure revealed presence of two loops with some important residues. The first loop, known as 380s loop, the mobility of which allows allosteric conformational changes after binding up of ADP-glucose with the enzyme thus allowing proper positioning of the substrate. The loop is found almost absent in SSIII with a meagre presence of 7 residues and lack of any helix. This could be a result of evolutionary functional divergence leading to structural adaptation for preferential binding to starch by three CBD domains and bringing the substrate in proximity of active site by interaction between CBDs and catalytic domain thus increasing substrate concentration at the site. The second loop was of 14 residues and comparatively less conserved. Dissection of Cis acting upstream regulatory region into its elements helps in the understanding of spatial, temporal and environmental influence on gene expression. Maize has got highest sequence similarity with the sorghum for the region as both have anatomical similarity which may require same mode of the gene expression. The core regulatory element like TATA box and CAAT box were identified in all the taxa under study except rice. The presence of TATA box, CAAT box and G box in maize was also reported [32]

## Supporting information

S1 Fig Alignment of dicots and monocots with the consensus sequence. Similar amino acids (SAA) in monocot are represented in grey colour while brown colour represents SAA in dicots. Deleted amino acids (DAA) in both monocots and dicots are represented in blank space on upper and lower side.
(TIF)

S2 Fig 3-D structure of the catalytic domain of SSIII protein in orthologs. (DOCX)

S1 Table Cis-elements identified in the promoter of *SSIII* gene. (DOCX)
