# Supplementary figures and images for: Conservation and divergence of Starch Synthase III genes of monocots and dicots
Source: PLoS One. 2017 Dec 14;12(12):e0189303. doi: 10.1371/journal.pone.0189303 (PMC5730167; doi:10.1371/journal.pone.0189303)

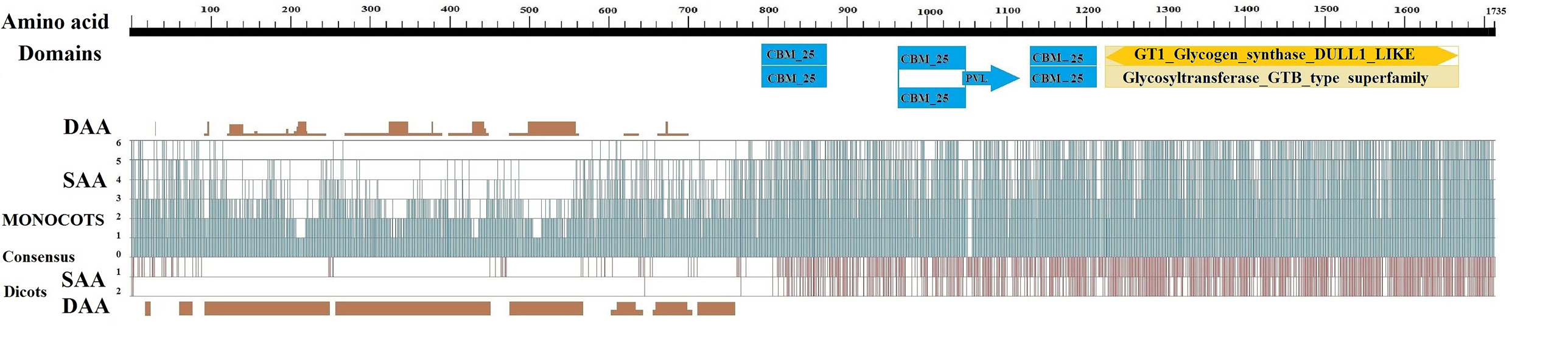

Supplement: S1 Fig — Similar amino acids (SAA) in monocot are represented in grey colour while brown colour represents SAA in dicots. Deleted amino acids (DAA) in both monocots and dicots are represented in blank space on upper and lower side. (TIF) [file pone.0189303.s001.tif]
