# Supplementary material for: Conservation and divergence of Starch Synthase III genes of monocots and dicots
Source: PLoS One. 2017 Dec 14;12(12):e0189303. doi: 10.1371/journal.pone.0189303 (PMC5730167; doi:10.1371/journal.pone.0189303)
Supplement: S2 Fig — (DOCX) [file pone.0189303.s002.docx]

Supp Fig. 1: 3-D structure of the catalytic domain of SSIII protein


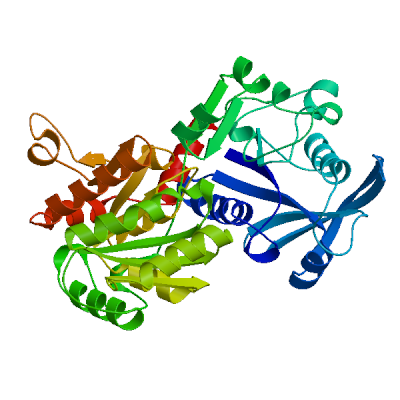

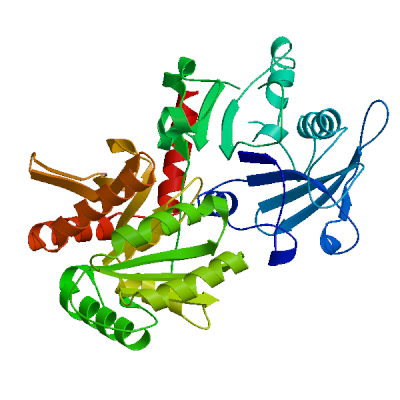


Wheat Maize


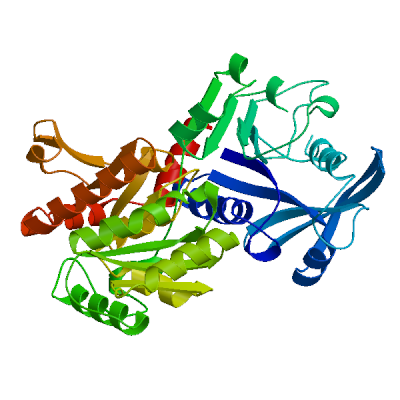

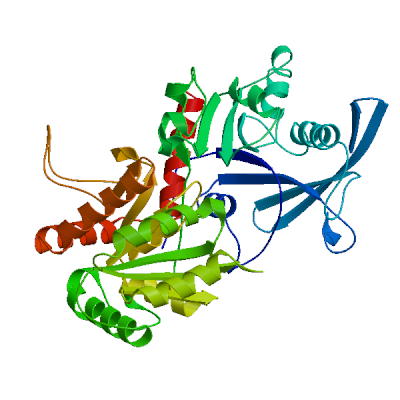


Barley Rice

**
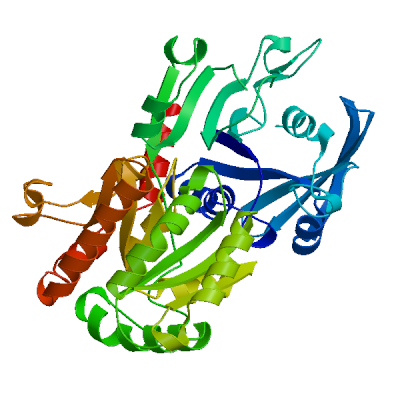

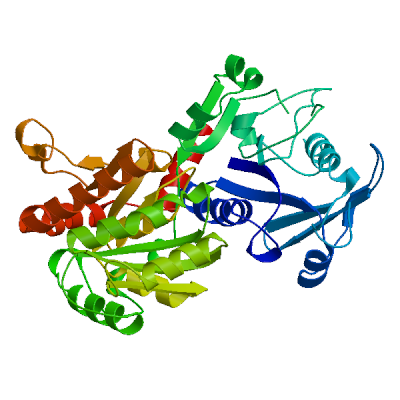
**

Sorghum Brachypodium

**
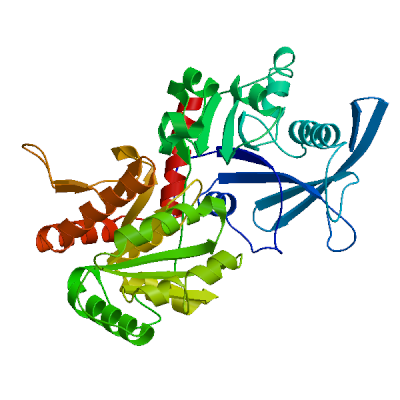

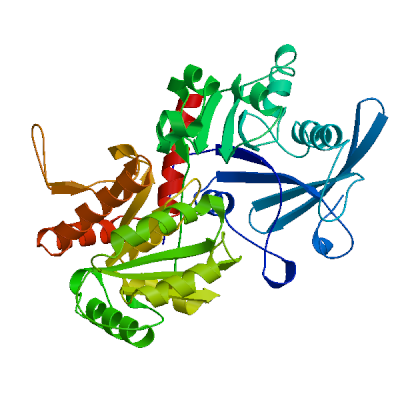
**

Arabidopsis Soybean
