# Supplementary material for: Conservation and divergence of Starch Synthase III genes of monocots and dicots
Source: PLoS One. 2017 Dec 14;12(12):e0189303. doi: 10.1371/journal.pone.0189303 (PMC5730167; doi:10.1371/journal.pone.0189303)
Supplement: S1 Table — (DOCX) [file pone.0189303.s003.docx]

Table 5. Cis-elements identified in the promoter of SSIII gene

| Element type | Motif sequence (5ʹ-3ʹ) | Function | Species | | | | | | | |
| --- | --- | --- | --- | --- | --- | --- | --- | --- | --- | --- |
|  |  |  | Wheat | Maize | Barley | *Brachypodium* | *Sorghum* | Rice | Soybean | *Arabidopsis* |
| Phytohormone  ABRE | CACGTG | cis-acting element involved in the abscisic acid responsiveness | - | + | - | + | + | + | - | + |
| ERE | ATTTCAAA | Ethylene-responsive element | + | _ | + | _ | _ | _ | _ | _ |
| Anaerobic induction  ARE | TGGTTT | cis-acting regulatory element essential for the anaerobic induction | + | + | + | + | - | + | - | + |
| Basal element  CAAT Box | CAAAT | common cis-acting element in promoter and enhancer regions | + | + | + | _ | + | _ | + | + |
|  | CCAAT |  | + | + | + | + | + | _ |  | + |
|  | CAATT |  | + | + | + | + | _ | _ | + | + |
|  | gGCAAT |  | _ | + | _ | + | _ | _ | _ | _ |
| CAT box | GCCACT | cis-acting regulatory element related to meristem expression | + | _ | + | + | _ | _ | _ | + |
| TATA-box | TAATA | core promoter element around -30 of transcription start | + | + | + | + | + | _ | + | + |
|  | TATAA |  | _ | + | _ | _ | + | _ | + | + |
|  | TACAAAA |  | + | + | + | _ | + | _ | _ | _ |
|  | TATA |  | + | + | + | + | + | _ | + | + |
|  | TTTTA |  | _ | + | + | + | + | _ | + | + |
|  | TATATAAA |  | _ | _ | _ | _ | + | _ | _ | _ |
|  | ATATAAT |  | _ | _ | _ | _ | + | _ | _ | + |
| Light  G-Box | CACGTT | cis-acting regulatory element involved in light responsiveness | + | + | + | _ | _ | _ | _ | _ |
|  | CACGTG |  | _ | + | _ | _ | + | _ | _ | + |
|  | CACACATGGAA |  | + | + | + | + | _ | _ | _ | _ |
|  | CACGAC |  | _ | + | _ | + | + | _ | _ | _ |
|  | CACGTT |  | _ | + | + | _ | _ | _ | _ | _ |
|  | CACGTC |  | _ | + | _ | _ | _ | _ | _ | _ |
|  | CACATGG |  | + | _ | + | + | _ | _ | _ | + |
|  | TGACACGTGGCTCT |  | _ | _ | _ | _ | _ | _ | _ | + |
| Spl | CC(G/A)CCC | light responsive element | + | _ | + | _ | + | _ | _ | _ |
|  | GGGCGG |  | + | _ | + | _ | _ | _ | _ | _ |
| Skn-1 motif | GTCAT | cis-acting regulatory element required for endosperm expression | _ | _ | + | _ | + | _ | _ | + |
| Chs-Unit 1 m1 | ACCTACCACAC | light responsive element | + | _ | + | _ | _ | _ | _ | _ |
| 5UTR Py-rich strech | TTTCTTCTCT | cis-acting element conferring high transcription levels | _ | _ | _ | + | + | + | + | _ |
| CCGTCC-box | CCGTCC | cis-acting regulatory element related to meristem specific activation | + | _ | + | + | _ | _ | _ | + |
| Heat stress  HSE | AAAAAATTTC | cis-acting element involved in heat stress responsiveness | + | _ | + | _ | _ | _ | _ | _ |
|  | CNNGAANNTTCNNG |  | + | _ | + | _ | _ | _ | _ | _ |
